# Design of a Humanoid Upper-Body Robot and Trajectory Tracking Control via ZNN with a Matrix Derivative Observer

**DOI:** 10.3390/biomimetics10080505

**Published:** 2025-08-02

**Authors:** Hong Yin, Hongzhe Jin, Yuchen Peng, Zijian Wang, Jiaxiu Liu, Fengjia Ju, Jie Zhao

**Affiliations:** School of Mechatronics Engineering, Harbin Institute of Technology, Harbin 150080, China; 19b908046@stu.hit.edu.cn (H.Y.); 23b908031@stu.hit.edu.cn (Y.P.); 24b308009@stu.hit.edu.cn (Z.W.); 22b308007@stu.hit.edu.cn (J.L.); 20b908029@stu.hit.edu.cn (F.J.); jzhao@hit.edu.cn (J.Z.)

**Keywords:** humanoid robot, upper-body design, trajectory tracking, zeroing neural network (ZNN), matrix derivative observer

## Abstract

Humanoid robots have attracted considerable attention for their anthropomorphic structure, extended workspace, and versatile capabilities. This paper presents a novel humanoid upper-body robotic system comprising a pair of 8-degree-of-freedom (DOF) arms, a 3-DOF head, and a 3-DOF torso—yielding a 22-DOF architecture inspired by human biomechanics and implemented via standardized hollow joint modules. To overcome the critical reliance of zeroing neural network (ZNN)-based trajectory tracking on the Jacobian matrix derivative, we propose an integration-enhanced matrix derivative observer (IEMDO) that incorporates nonlinear feedback and integral correction. The observer is theoretically proven to ensure asymptotic convergence and enables accurate, real-time estimation of matrix derivatives, addressing a fundamental limitation in conventional ZNN solvers. Workspace analysis reveals that the proposed design achieves an 87.7% larger total workspace and a remarkable 3.683-fold expansion in common workspace compared to conventional dual-arm baselines. Furthermore, the observer demonstrates high estimation accuracy for high-dimensional matrices and strong robustness to noise. When integrated into the ZNN controller, the IEMDO achieves high-precision trajectory tracking in both simulation and real-world experiments. The proposed framework provides a practical and theoretically grounded approach for redundant humanoid arm control.

## 1. Introduction

Humanoid robots, owing to their human-like morphology, are gaining increasing popularity across a wide range of application scenarios. Their anthropomorphic design facilitates intuitive interaction and task adaptability, making them suitable for both service and industrial environments. Typical applications include assisted dressing, tool operation, disaster response, and door opening [1,2,3,4]. Within these contexts, the upper-body components of humanoid robots serve as primary interfaces for manipulation and interaction. Consequently, the structural design and functional integration of humanoid upper-body systems play critical roles in achieving efficient, effective, and versatile task execution.

Several advanced humanoid platforms have demonstrated remarkable capabilities. Robonaut 2, developed by NASA, has been deployed on the International Space Station, successfully performing panel manipulation and cleaning tasks, thereby confirming its utility in microgravity conditions [5]. Justin, developed by the German Aerospace Center (DLR), is recognized for its dexterity, showcasing complex manipulation tasks such as emptying wastebaskets, serving beverages, and catching flying objects [6]. The HUBO robot, designed by the Korea Advanced Institute of Science and Technology (KAIST), features 41 degrees of freedom (DOFs) and excelled in challenging tasks such as vehicle operation and ladder climbing during the DARPA Robotics Challenge [7]. The ARMAR series from the Karlsruhe Institute of Technology incorporates wheeled mobile bases and emphasizes effective human–robot interaction combined with upper-limb manipulation [8]. Kawada Industries’ HRP-5P, an evolution of earlier HRP models, employs robust, high-range joints suitable for construction and heavy-load tasks, convincingly performing fully autonomous drywall installation [9]. SURENA IV was developed as a cost-effective anthropomorphic robot designed for practical applications, capable of drilling, visual servoing-based object relocation, and even handwriting tasks [10]. The BHR-6 humanoid robot from Beijing Institute of Technology offers outdoor capabilities, including walking, rolling, and self-protective falling, owing to its 23 DOFs and robust mechanical design [11]. JET, tailored for industrial and service sectors, simplifies maintenance through modular electronics and enhanced mechanical design, effectively expanding its operational workspace [12]. TOCABI introduces advanced torque-controlled mechanisms, enabling agility and whole-body manipulation comparable to human adults through back-drivable joints [13]. BIT-DMR integrates a humanoid dual-arm upper body onto a tracked mobile platform, demonstrating versatility in rescue scenarios involving payload management, obstacle removal, and casualty evacuation [14]. FC-EODR, a dual-arm explosive ordnance disposal robot, has successfully executed tasks such as remote wire-cutting and screw-tightening, demonstrating reliability in critical environments [15]. The ergonomically optimized iCub 3/ergoCub robot features a design that minimizes metabolic costs during collaborative tasks, enabling effective manipulation of payloads up to 10 kg [16].

Parallel to advancements in hardware, significant progress has been achieved in the development of robust control algorithms. Among these methods, the zeroing neural network (ZNN), a subclass of recurrent neural networks (RNNs), has emerged as a particularly powerful tool for real-time control. The rationale for its selection lies in its nature as a model-driven dynamical system, specifically engineered for the online solution of time-varying problems. Unlike conventional neural networks that act as function approximators, ZNNs function as specialized dynamic equation solvers, making them inherently suitable for tasks like real-time trajectory tracking that demand guaranteed error convergence.

The structure of a ZNN is elegantly simple, defined not by layers of neurons but by its governing differential equation. The design principle involves constructing a dynamic evolution, typically of the form $\dot{e}\left(t\right)=-\lambda f\left(e\left(t\right)\right)$ [17], where a problem-specific error e(t) is driven to zero. The choice of the activation function f(·) determines the type of convergence (e.g., exponential, finite-time, fixed-time), and this dynamic equation constitutes the essence of the ZNN solver.

This model-driven approach leads to a crucial and distinguishing advantage: ZNNs are “training-free.” Their stability is mathematically proven, often using Lyapunov theory, and their performance is tuned via design parameters (e.g., a gain coefficient) rather than learned weights. This “training-free” characteristic makes ZNNs exceptionally well-suited for applications where continuous adaptation and immediate deployment without extensive data collection are required.

Building on this foundational concept, ZNNs have been extensively employed to address key challenges in robotic manipulator trajectory tracking. Research in this area can be broadly categorized into two main thrusts: (1) online estimation of time-varying matrix pseudo-inverses and (2) solving quadratic programming (QP) formulations for constrained trajectory tracking tasks.

In addressing the first challenge, Zhang et al. [17] initially proposed the ZNN framework, which utilized a matrix estimation error function and its derivative to achieve exponential convergence. Subsequently, this framework was generalized to calculate the time-varying full-rank matrix Moore–Penrose inverse (i.e., pseudoinverse) [18]. Subsequent research efforts aimed at enhancing convergence speed and robustness included the incorporation of adaptive time-varying parameters [19]. Furthermore, specialized nonlinear activation functions were designed to guarantee prescribed-time convergence [20,21], and fixed-time convergence models for non-square matrix pseudoinversion were developed [22,23]. To improve noise tolerance, an integral-enhanced ZNN (IEZNN) that ensures asymptotic stability and adjustable residual bounds under measurement disturbances was introduced [24]. Exponential and finite-time convergence IEZNN models were also presented for enhanced robustness [25,26,27]. The feasibility of the ZNN models mentioned above in trajectory tracking control of redundant manipulators or mobile manipulators has been verified through simulations.

The second challenge focuses on managing joint-limit and obstacle constraints through QP formulations. In this regard, joint-velocity and position constraints have been reformulated into equality conditions using slack variables, allowing real-time solutions via ZNN [28,29]. A unified QP framework has been proposed to support cyclic motion planning under physical constraints, with prescribed-time ZNN solvers ensuring convergence [30]. Beyond slack variables, alternative QP formulations involving auxiliary state variables have been introduced and implemented using corresponding ZNN-based solvers [31]. Moreover, methods based on Lagrange multipliers and Karush–Kuhn–Tucker (KKT) conditions have been employed to handle comprehensive joint constraints, and their recurrent neural dynamics have been successfully validated on both single- and multi-robot platforms [32].

However, despite these significant advances in both humanoid hardware and ZNN-based control theory, a critical gap separates theoretical models from practical implementation. First, as summarized in Table 1, many existing humanoid platforms face constraints in workspace and dexterity, limiting their effectiveness in complex bimanual tasks. Second, and more critically, the practical deployment of ZNN-based controllers faces a fundamental barrier: their dependence on the real-time availability of matrix time derivatives (e.g., the Jacobian derivative). This information is notoriously difficult to obtain in physical systems, which critically compromises controller stability and precision.

To address these challenges, this paper makes a two-fold contribution. We first introduce a novel humanoid upper-body robotic system with enhanced DOFs and a modular hollow joint design utilizing resolver feedback for improved performance. In parallel, we develop a high-precision, integration-enhanced matrix derivative observer with nonlinear feedback mechanisms. This observer directly tackles the aforementioned limitation, significantly improving the robustness and real-time performance of ZNN-based trajectory tracking frameworks and bridging the gap between theoretical ZNN models and their practical implementation.

The main contributions of this paper are summarized as follows:

1. A 22-DOF humanoid upper-body robotic system is presented, consisting of a pair of 8-DOF arms, a 3-DOF torso, and a 3-DOF head. The design is inspired by human biomechanics. Workspace analysis demonstrates that the proposed system exhibits superior kinematic performance.
2. A cost-effective, interference-resistant hollow joint module based on resolver feedback is developed. Compared with traditional encoder-based solutions, this design offers improved electromagnetic immunity and modular scalability, making it well-suited for highly redundant robotic systems.
3. To overcome the reliance on unavailable Jacobian derivatives in the existing ZNN literature, an integration-enhanced matrix derivative observer (IEMDO) is proposed. The observer ensures asymptotic convergence and significantly enhances numerical stability and real-time feasibility of ZNN-based inverse kinematics solutions.
4. The proposed IEMDO is embedded into the ZNN control framework and implemented on the developed humanoid platform. Both simulation and physical experiments validate the effectiveness, robustness, and practical feasibility of the proposed system and algorithm.

The remainder of this paper is organized as follows: Section 2 presents the design of the humanoid upper-body robotic system, including both the mechanical structure and electrical components. Section 3 introduces the ZNN method for trajectory tracking control, discusses its reliance on the Jacobian matrix derivative, and proposes a novel matrix derivative observer with a theoretical analysis of its asymptotic convergence. In Section 4, simulation studies are conducted to evaluate the kinematic workspace of the proposed robot, verify the accuracy of the matrix derivative observer, and demonstrate its integration into the ZNN framework for trajectory tracking. Section 5 implements the proposed control scheme on the physical humanoid robotic platform to validate its practical effectiveness. Finally, Section 6 concludes the paper and outlines future research directions.

## 2. Mechanical and Electrical System Design

### 2.1. Overview of Upper-Body Mechanical Subsystems

The human upper body typically comprises the head, two arms, and the torso. Accordingly, the upper-body design of the humanoid robot is divided into three subsystems—a head subsystem, a dual-arm subsystem, and a torso subsystem—which are developed separately and then integrated into the complete humanoid robot. Because this work focuses on manipulative capability, the design sequence begins with the dual-arm subsystem to emphasize and enhance the robot’s dexterous manipulation. The torso subsystem is then designed according to the payload and other requirements imposed by the arms, and finally the head subsystem is developed to broaden the camera’s field of view for target acquisition.

#### 2.1.1. Redundant Dual-Arm Mechanism

Bimanual tasks often involve complex object-manipulation skills—such as opening a bottle, folding laundry, or tying cables—that are simply unattainable with a single arm. Owing to their greater versatility and human-like workspace, dual-arm robots are viewed as better suited for manipulation in unstructured settings, including small-batch production lines and household environments.

**Figure 1:**
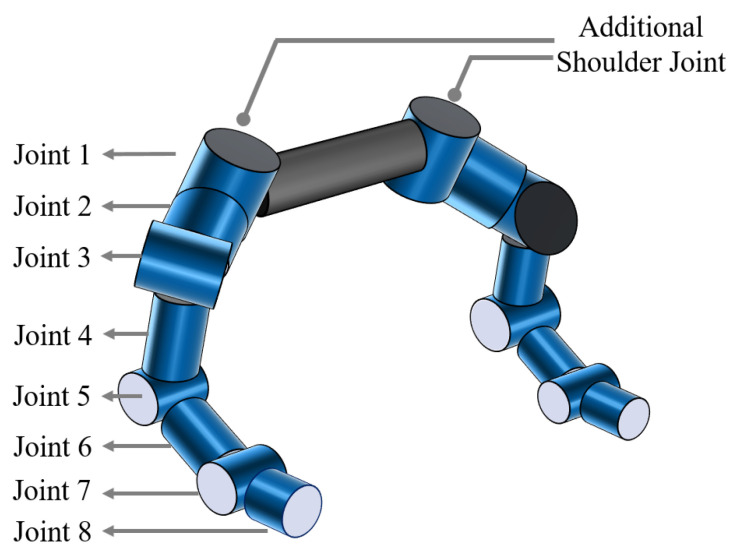
Dual-arm subsystem.

To endow our dual-arm system with superior kinematic performance, we draw inspiration from the human sternoclavicular (shoulder–chest) joint and propose an eight-DOF redundant manipulator for each arm, as illustrated in Figure 1. The additional shoulder joint enables lateral translation of the shoulder, allowing both arms to maintain excellent kinematic dexterity in the primary workspace in front of the torso while still preserving the ability to perform single-arm tasks when required.

#### 2.1.2. Three-DOF Torso Design

Human trunk motion is produced by waist muscles acting on the pelvic–hip complex, allowing flexion/extension, lateral bending, and axial rotation, thereby greatly enhancing mobility and dexterity. To emulate these capabilities, the humanoid robot’s waist is endowed with three degrees of freedom. A movable torso enlarges the visual tracking range of the head-mounted vision system, increases kinematic redundancy, and augments overall manipulation ability. To maximize the workspace of the dual-arm system while maintaining high dexterity, the proposed waist adopts a serial architecture with three independently driven joints.

#### 2.1.3. Vision-Driven Head Assembly

**Figure 2:**
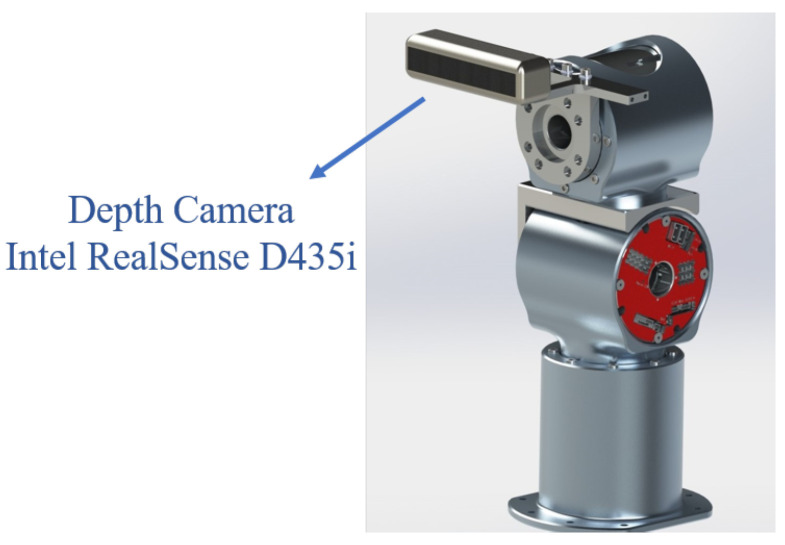
Head subsystem.

In humans, the neck muscles provide pitch and yaw motion of the head, assisting the visual system in capturing targets. Accordingly, the robot head requires at least two DOFs (pitch and yaw) to acquire and track specific objects. In addition, when the human line of sight is blocked, a roll (lateral tilt) motion is often used to peer around obstacles without bending the torso. To reproduce this function, the robot head is given three DOFs—pitch, yaw, and roll—allowing rapid target tracking and basic visual obstacle avoidance. A camera mounted on the head supplies the robot with target pose information, based on the camera parameters and installation geometry, for subsequent state estimation and manipulation. The head mechanism is implemented as a serial joint chain and enclosed in a humanoid shell for aesthetic reasons (Figure 2). When the field of view is insufficient, torso motion can be used to reposition the head base and extend the visual range.

#### 2.1.4. Resolver-Based Hollow Joint Modules

The dual-arm subsystem provides 16 DOFs and the torso 3 DOFs, all realized in serial chains. To simplify the design and ease maintenance, 19 modular hollow joints with an identical mechanical principle have been developed. Each joint module consists of a permanent-magnet synchronous motor, a harmonic reducer, a resolver, and a resolver-to-digital conversion board. Fail-safe brakes manufactured by Miki Pulley (Japan) are installed on the high-load torso joints; they are controlled via the drive I/O ports and lock the motor shaft upon power loss, ensuring safe emergency stops and preventing the upper-body robot from overturning. Thus, the joint modules provide both precise motion control and essential safety functions.

**Figure 3:**
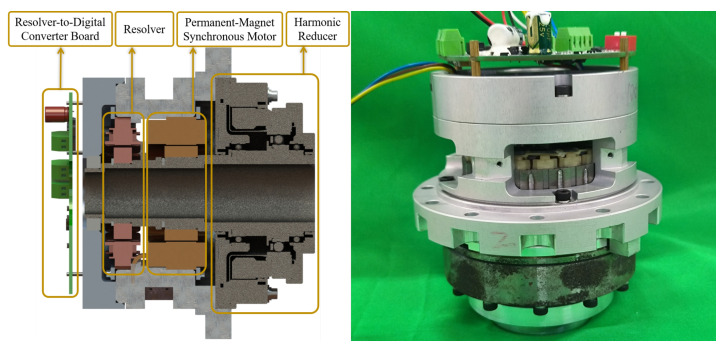
Cross-section and physical view of the joint module structure.

Depending on the load requirements, the motors are selected from the KMF series frameless PMSMs (models 60AL, 76AL, and 76BL) produced by Dongmao Industrial Equipment. The harmonic drives are LHSG-17, LHSG-25, and LHSG-32 series units from Suzhou Green Harmonic. The resolvers are leaded reluctance types (26 mm and 52 mm) supplied by Suzhou Delta, and the resolver interface is implemented on a custom PCB. A cross-section of the joint module is shown in Figure 3.

### 2.2. Electrical Design

The humanoid upper-body robot adopts a central controller–joint drive architecture. A Beckhoff C6930-0060 industrial PC is used as the main controller, and each joint is driven by an Elmo Gold Solo servo drive. These mature commercial products ensure high reliability of the control scheme.

**Figure 4:**
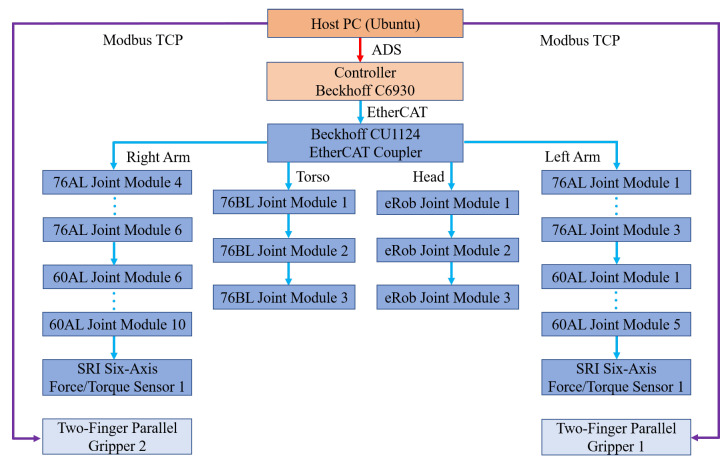
Communication architecture of the humanoid upper-body robot.

Communication. EtherCAT is employed as the primary fieldbus. The Beckhoff controller acts as the EtherCAT master, exchanging data with the Elmo drives and the six-axis force/torque sensor mounted on the dual-arm end effectors, which serve as slave devices. A host PC running Linux communicates with the gripper via Modbus TCP and exchanges data with the Beckhoff controller through the ADS protocol, thereby completing the overall communication framework of the robot (see Figure 4).

**Figure 5:**
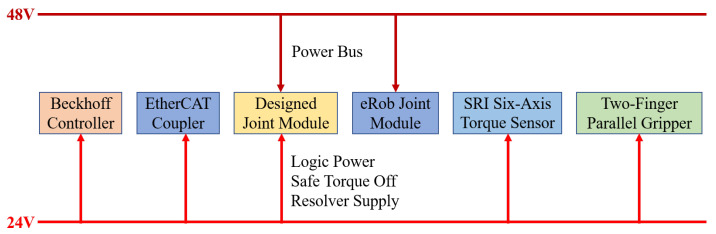
Power distribution scheme of the humanoid upper-body robotic system.

Power supply. The servo drives, main controller, end-effector gripper, six-axis force sensor, and resolver decoding circuits in each joint are powered at 24 V, whereas the motors are supplied at 48 V. Consequently, the power architecture comprises a 48 V power bus for actuation and a 24 V auxiliary bus for control electronics, as illustrated in Figure 5.

### 2.3. System Integration and Kinematic Parameter Setup

Building on the joint modules, drives, and controller specified in the previous subsections—and adhering to the anthropomorphic design principles for the head, torso, and dual arms—we have completed the overall design of the humanoid upper-body robot.

For the arm links, surface-modeling techniques were employed to align the joints along a common axis, ensuring that the upper body conforms to biomechanical principles. Stress analysis, topology optimization, and shape optimization were carried out to keep the structure compact while satisfying strength requirements and providing sufficient space for cable routing.

**Figure 6:**
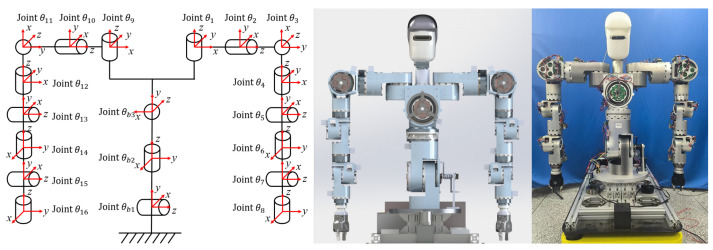
Kinematic diagram and prototype of the humanoid upper-body robot.

The resulting three-dimensional model of the humanoid upper-body robot and the assembled experimental platform are shown in Figure 6. The corresponding Denavit-Hartenberg (D-H) parameters are listed in Table 2.

## 3. Trajectory Tracking via ZNN with Jacobian Derivative Observer

### 3.1. Problem Formulation and ZNN Framework

In practical robot manipulator trajectory tracking tasks, joint angle and velocity constraints must be explicitly considered. Extending the control strategy presented in [33], the trajectory tracking problem with constraints can be formulated as solving the following time-varying linear equation system:

(1) $$\left\{\begin{array}{cc} \; & J\left(\theta \left(t\right)\right)\dot{\theta }\left(t\right)={\dot{r}}_{d}\left(t\right)-J_{b}\left(\theta_{b}\left(t\right)\right){\dot{\theta }}_{b}\left(t\right)\\ \; & {\dot{\theta }}^{-}\leq \dot{\theta }\left(t\right)\leq {\dot{\theta }}^{+}\\ \; & \theta^{-}\leq \theta \left(t\right)\leq \theta^{+} \end{array}\right.$$

where $\theta \left(t\right)\in R^{16}$ and $\dot{\theta }\left(t\right)\in R^{16}$ represent joint angles and velocities respectively, $J\left(\theta \left(t\right)\right)\in R^{6\times 16}$ denotes the dual-arm Jacobian, and ${\dot{r}}_{d}\left(t\right)$ is the desired end-effector velocity. The torso motion is predefined by $\theta_{b}\left(t\right)\in R^{3}$ with corresponding Jacobian $J_{b}\left(\theta_{b}\left(t\right)\right)\in R^{6\times 3}$. The constraints ${\dot{\theta }}^{\pm }$ and $\theta^{\pm }$ define velocity and angle limits.

Inspired by the approach in [29], the constrained system (1) is solved using the following ZNN model:

(2) $$\dot{u}\left(t\right)=-A^{†}\left(t\right)\left(B\left(t\right)u\left(t\right)-\dot{q}\left(t\right)\right)+\lambda F\left(W(t)u(t)-q(t)\right)$$

where λ>0 is a design parameter and F(·) is a monotonically increasing odd activation function array. Matrices and vectors involved in Equation (2) are defined as follows:

(3) $$\begin{array}{cc} A(t) & =\left[\begin{array}{cc} J(t) & 0\\ C(t) & 2D(t) \end{array}\right]\in R^{38\times 48},\;B\left(t\right)=\left[\begin{array}{cc} \dot{J}\left(t\right) & 0\\ \dot{C}\left(t\right) & 0 \end{array}\right]\in R^{38\times 48},\;C\left(t\right)=\left[\begin{array}{c} -I\\ I \end{array}\right]\in R^{32\times 16}\\ W(t) & =\left[\begin{array}{cc} J(t) & 0\\ C(t) & D(t) \end{array}\right]\in R^{38\times 48},\;d\left(t\right)=\left[\begin{array}{c} -\max\left\{{\dot{\theta }}^{-},\;\mu \left(\theta^{-}-\theta \left(t\right)\right)\right\}\\ \min\left\{{\dot{\theta }}^{+},\;\mu \left(\theta^{+}-\theta \left(t\right)\right)\right\} \end{array}\right]\in R^{32}\\ u(t) & ={\left[{\dot{\theta }}^{T}\left(t\right),\;y^{T}\left(t\right)\right]}^{T}\in R^{48},\;q\left(t\right)={\left[{\left({\dot{r}}_{d}\left(t\right)-J_{b}\left(t\right){\dot{\theta }}_{b}\left(t\right)\right)}^{T},\;d^{T}\left(t\right)\right]}^{T}\in R^{38} \end{array}$$

where $y\left(t\right)\in R^{32}$ is the slack variable, $D\left(t\right)=\mathrm{diag}[y_{1}\left(t\right),\ldots ,y_{32}\left(t\right)]$ is the corresponding diagonal matrix, $I\in R^{16\times 16}$ is the unit matrix, μ>0 is the designed parameter.

As shown in Equations (2) and (3), the ZNN model requires the time derivative of the Jacobian matrix, whose acquisition poses significant challenges. Analytical computation is often intractable for high-dimensional redundant systems, while numerical differentiation suffers from noise sensitivity and poor stability. Moreover, the large size and complex structure of J(t) incur substantial computational overhead when directly computing $\dot{J}\left(t\right)$. These limitations highlight the need for a stable, efficient, and convergent matrix derivative observer that enables accurate real-time estimation under practical conditions.

### 3.2. Matrix Derivative Observer Design

Given a time-varying matrix $M\left(t\right)\in R^{m\times n}$, an effective estimate of its time derivative $\dot{M}\left(t\right)$ is sought by treating M(t) as the state output of an implicit dynamic system. The following state equation is introduced:

(4) $$\dot{M}\left(t\right)=S\left(t\right)$$

where $S\left(t\right)≜\left\{s_{ij}\left(t\right)\right\}\in R^{m\times n}$ denotes the true derivative of M(t). Let $\hat{M}≜\left\{{\hat{m}}_{ij}\right\}$ and $\hat{S}≜\left\{{\hat{s}}_{ij}\right\}$ be the estimated state and its derivative, with observation errors defined as

(5) $$\tilde{M}=M-\hat{M}≜\left\{{\tilde{m}}_{ij}\right\},\;\tilde{S}=S-\hat{S}≜\left\{{\tilde{s}}_{ij}\right\}$$

Based on this modeling, the following observer structure is proposed:

(6) $$\dot{\hat{M}}=\hat{S}+\gamma \Sigma \left(\tilde{M}\right)$$

where γ>0∈R is a gain parameter and $\Sigma \left(\tilde{M}\right)≜\left\{\sigma \left({\tilde{m}}_{ij}\right)\right\}$ is a component-wise activation function array. Each element σ(·) is a monotonically increasing activation function.

From Equations (4)–(6), the error dynamics are given by

(7) $$\dot{\tilde{M}}=-\gamma \Sigma \left(\tilde{M}\right)+\tilde{S}$$

To assess stability, the ideal case $\tilde{S}\equiv 0_{m\times n}$ is considered. It is evident that the origin $\tilde{M}=0_{m\times n}$ serves as the unique equilibrium. A Lyapunov candidate function is selected as

(8) $$V=\mathrm{tr}({\tilde{M}}^{T}\tilde{M})/2$$

with its time derivative computed as

(9) $$\dot{V}=-\gamma \mathrm{tr}\left({\tilde{M}}^{T}\Sigma \left(\tilde{M}\right)\right)\leq 0$$

According to LaSalle’s invariance principle, the system (7) is asymptotically stable at the origin. For bounded disturbances $\tilde{S}$, system (7) exhibits input-to-state boundedness, i.e., $\tilde{M}\left(t\right)$ remains bounded and converges as $\tilde{S}\to 0_{m\times n}$.

*Remark:* The term $\Sigma (\tilde{M})$ functions as a nonlinear spring, introducing damping and promoting stabilization. When $\hat{S}\equiv 0_{m\times n}$ and γ≫1, the approximation $\dot{\tilde{M}}\approx 0_{m\times n}$ holds, implying that $\dot{M}\approx \dot{\hat{M}}$. Although $\hat{S}$ is omitted, the derivative can still be approximated through high-gain feedback. Nonetheless, excessively large gains may amplify noise and compromise stability and are therefore discouraged.

To further enhance observer accuracy and robustness, the error $\tilde{S}$ is required to approach zero. Since S(t) is unobservable, a direct cost function is impractical. Instead, $\tilde{M}$ serves as an indirect indicator of performance. A new cost function is defined as

(10) $$V=\mathrm{tr}(Z^{T}Z)/2$$

where

(11) $$Z=\tilde{M}+\gamma \int_{0}^{t}\Sigma \left(\tilde{M}\right)d\tau$$

Convergence of Z to zero implies that $\tilde{M}$ and its integral term vanish, thereby indirectly driving $\tilde{S}\to 0_{m\times n}$. Taking the derivative of Equation (10) and applying Equation (7) yields

(12) $$\dot{V}=\mathrm{tr}\left(Z^{T}\left(S-\hat{S}\right)\right)$$

A feedback law for $\hat{S}$ is defined as

(13) $$\hat{S}=\alpha Z+\beta \Phi \left(Z\right)$$

where α,β>0∈R are tuning parameters. The mapping $\Phi \left(Z\right)≜\left\{\phi \left[z_{ij},\varepsilon \left(t\right)\right]\right\}$ is defined in an element-wise manner, with the auxiliary parameter ε(t) satisfying $\lim_{t\to \infty }\varepsilon (t)=0$. The detailed expression of the function ϕ(·) is defined as

(14) $$\phi \left[z_{ij},\varepsilon \left(t\right)\right]=\frac{z_{ij}}{|z_{ij}|+\epsilon \left(t\right)},\;\varepsilon \left(t\right)=\rho e^{-0.001t}$$

Substituting into Equation (6) yields the improved observer formulation

(15) $$\dot{\hat{M}}=\alpha Z+\beta \Phi \left(Z\right)+\gamma \Sigma \left(\tilde{M}\right)$$

In conclusion, a matrix derivative observer is developed based on dynamic neural network theory. Convergence and robustness are ensured through the use of nonlinear state feedback Σ(·) and an integral-enhanced term Z, enabling indirect suppression of unmeasurable disturbances $\tilde{S}$.

Therefore, the proposed observer is named the integral-enhanced matrix derivative observer (IEMDO).

### 3.3. Theoretical Analysis

**Theorem** **1.** *Let the matrix $M\left(t\right)≜m_{ij}\left(t\right)$ consist of smooth time-varying elements, and assume that its derivative $S\left(t\right)≜s_{ij}\left(t\right)$ is bounded element-wise. If the parameter β in the derivative observer (15) satisfies $\beta \geq \max|s_{ij}\left(t\right)|$, then the cost function V defined in Equation (10) converges asymptotically to zero. Consequently, the matrix Z, the estimation error $\tilde{M}$, and its time derivative $\dot{\tilde{M}}$ all asymptotically converge to the zero matrix $0_{m\times n}$.*

**Proof of Theorem** **1.** Substituting Equation (13) into Equation (12), the time derivative of *V* is obtained as

(16) $$\dot{V}=-\alpha \mathrm{tr}\left(Z^{T}Z\right)+\mathrm{tr}\left(Z^{T}S\right)-\beta \mathrm{tr}\left[Z^{T}\Phi \left(Z\right)\right]$$

By expanding the trace expressions element-wise, the last two terms are rewritten as

(17) $$\mathrm{tr}\left(Z^{T}S\right)-\beta \mathrm{tr}\left(Z^{T}\Phi \left(Z\right)\right)=\sum_{i=1}^{m}\sum_{j=1}^{n}\left\{z_{ij}s_{ij}-\beta z_{ij}\phi \left[z_{ij},\varepsilon \left(t\right)\right]\right\}$$

Given the condition $\beta \geq |s_{ij}\left(t\right)|$ and the properties of ϕ(·), it follows that

(18) $$z_{ij}s_{ij}-\beta z_{ij}\phi \left[z_{ij},\varepsilon \left(t\right)\right]\leq \left(|s_{ij}|-\beta \right)|z_{ij}||\phi \left[z_{ij},\varepsilon \left(t\right)\right]\left|+\varepsilon \left(t\right)\right|s_{ij}||\phi \left[z_{ij},\varepsilon \left(t\right)\right]\left|\leq \varepsilon \left(t\right)\right|s_{ij}|$$

Thus, an upper bound for the trace difference is established:

(19) $$\mathrm{tr}\left(Z^{T}S\right)-\beta \mathrm{tr}\left(Z^{T}\Phi \left(Z\right)\right)\leq \varepsilon \left(t\right)\sum_{i=1}^{m}\sum_{j=1}^{n}\left|s_{ij}\right|≜\delta \left(t\right)$$

Substituting into Equation (16), the following inequality is derived:

(20) $$\dot{V}\leq -2\alpha V+\delta \left(t\right)$$

Since ε(t)→0 as t→∞ and $s_{ij}\left(t\right)$ are bounded, δ(t) vanishes asymptotically. By the comparison lemma, it is ensured that V(t) converges asymptotically to zero.

To determine whether $\tilde{S}\to 0_{m\times n}$, the integral representation $Z\left(t\right)=\int_{0}^{t}\dot{Z}\left(\tau \right)d\tau$ is considered. By Barbalat’s Lemma, convergence of $\dot{Z}$ to zero follows from uniform continuity and boundedness of Z. Since $\dot{Z}=\tilde{S}$, the uniform continuity of $\dot{Z}$ is ensured by the smoothness of M(t) and continuity of $\hat{S}$ via Equation (13). Given V→0, it follows that $Z\to 0_{m\times n}$, thus confirming the uniform continuity.

Therefore, $\dot{Z}\to 0_{m\times n}$, implying $\tilde{S}\to 0_{m\times n}$. From Equation (7), this leads to the asymptotic convergence of $\tilde{M}$. Additionally, $\dot{\tilde{M}}\to 0_{m\times n}$ follows by substitution. This completes the proof. □

## 4. Simulation

To validate the proposed humanoid upper-body robot and the matrix derivative observer under the ZNN framework, a sequence of simulation studies is presented to assess both kinematic performance and control effectiveness. First, a comprehensive workspace evaluation is carried out for the 22-DOF robotic system, benchmarking it against configurations inspired by existing well-known humanoid platforms. Subsequently, the estimation accuracy and noise robustness of the proposed matrix derivative observer (IEMDO) are assessed using a benchmark high-dimensional matrix, and its performance is compared against the conventional finite-difference method. Finally, the observer is embedded into a ZNN-based trajectory tracking controller for the designed humanoid upper-body robot.

### 4.1. Kinematic Workspace Evaluation and Comparative Analysis

To assess the reachability and spatial performance of the proposed humanoid upper-body robot, a comprehensive kinematic workspace analysis is conducted using the Monte Carlo method. Feasible joint configurations are sampled to project the corresponding end-effector positions into Cartesian space. To quantitatively benchmark our design against established architectures, four configurations are evaluated to isolate the effects of torso motion and the additional shoulder degrees of freedom (DOFs):

- **Configuration 1 (Proposed Design)**: The complete 22-DOF model, featuring a 3-DOF torso and dual 8-DOF arms.
- **Configuration 2 (Typical 7-DOF Arms with Torso)**: A 20-DOF model without the additional shoulder joints, representing advanced platforms that combine a torso with standard 7-DOF arms, such as Justin [6] and iCub3 [16].
- **Configuration 3 (8-DOF Arms, Fixed Torso)**: A 19-DOF model without torso joints, isolating the contribution of the 8-DOF arm design.
- **Configuration 4 (Baseline: 7-DOF Arms, Fixed Torso)**: A 17-DOF model without both torso and additional shoulder joints, representing a baseline configuration common to many classic humanoids like ASIMO or early-generation platforms.

To quantify performance, the product of total and common workspace areas (PTCWAs) is adopted as a performance index:

(21) $$\mathrm{PTCWA}=V_{\mathrm{total}}\times V_{\mathrm{common}}$$

where $V_{\mathrm{total}}$ and $V_{\mathrm{common}}$ represent the total and overlapping reachable areas of the two arms, respectively. This metric is widely used to characterize the balance between individual arm reach and coordinated bimanual capability [34].

**Figure 7:**
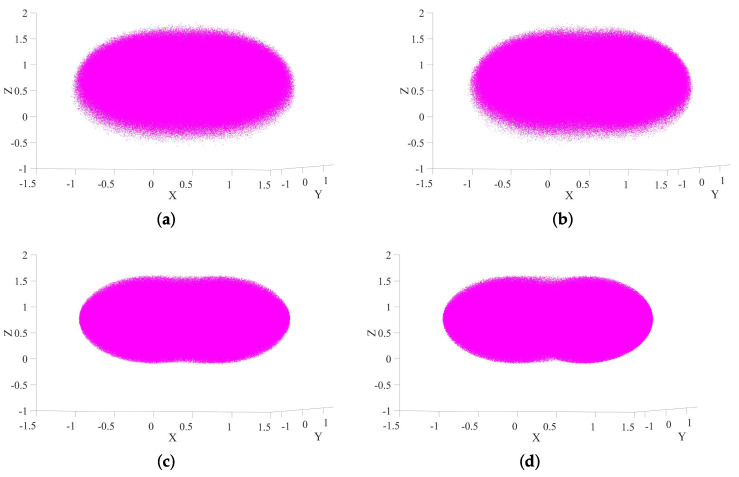
Comparison of total workspaces under different configurations. (**a**) Configuration 1. (**b**) Configuration 2. (**c**) Configuration 3. (**d**) Configuration 4.

**Figure 8:**
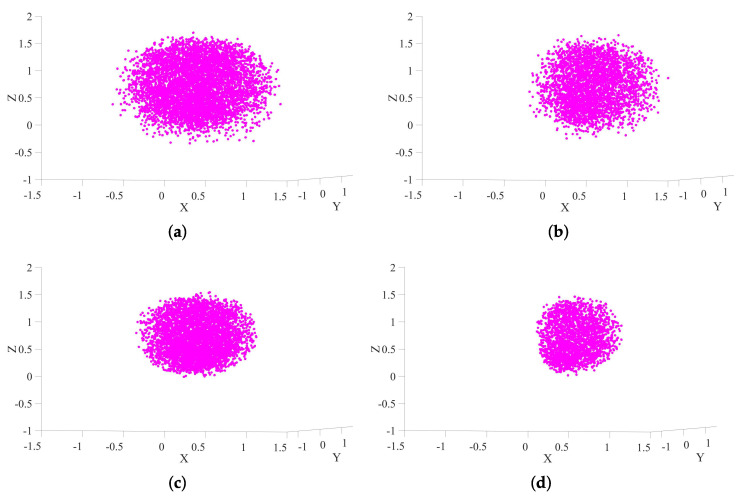
Comparison of common workspaces under different configurations. (**a**) Configuration 1. (**b**) Configuration 2. (**c**) Configuration 3. (**d**) Configuration 4.

The spatial distributions of the total and common workspaces for all four configurations are visualized in Figure 7 and Figure 8, respectively. The quantitative results, summarized in Table 3, provide a clear and compelling demonstration of the performance gains achieved through our bio-inspired design.

Our analysis begins with the baseline (Configuration 4), which represents a conventional 7-DOF dual-arm system on a fixed base. From this starting point, we can isolate the contributions of each design enhancement. The introduction of a 3-DOF torso alone (Configuration 3 vs. 4) expands the common workspace by a remarkable 109.2%, underscoring the critical role of torso mobility in creating a functional bimanual manipulation area. This finding aligns with and extends upon previous studies on torso-driven manipulation [35]. Separately, upgrading from 7-DOF to 8-DOF arms by adding shoulder translation joints (comparing Configuration 2 to a hypothetical fixed-torso baseline) yields an even more dramatic 223.9% increase in common workspace, highlighting the profound impact of this specific redundant joint on dexterous bimanual tasks.

When these enhancements are combined, our proposed design (Configuration 1) achieves a total common workspace of 5.3398 m^3^, representing a remarkable 368.2% expansion compared to the baseline. While the total workspace also sees a significant increase of 87.7%, the exponential growth of the common workspace is the key indicator of superior dual-arm coordination capacity. This result is not merely a numerical improvement; it directly translates to the robot’s ability to perform complex, human-like tasks such as manipulating large objects or turning a steering wheel, which are often unattainable for platforms with limited workspace overlap.

Moreover, these enhancements are not merely theoretical. The significantly expanded common workspace is essential for practical bimanual tasks such as object transfer, turning a wheel, or manipulating large tools—operations that require both arms to function cooperatively within a shared reachable region. These types of tasks have already been implemented by our group in related studies using the proposed humanoid robotic platform [33,36,37]. In contrast, robots such as ASIMO and iCub3, while effective for mobility or perceptual research, often lack sufficient workspace overlap to perform such coordinated dual-arm operations.

Additionally, this substantial increase in workspace directly translates to enhanced operational flexibility. In cluttered environments like households or industrial workstations, the robot can perform complex manipulation tasks over a larger area without needing to reposition its base, significantly improving both task efficiency and operational safety.

### 4.2. Validation and Comparative Analysis of the Matrix Derivative Observer

A key motivation for developing the IEMDO is to address the practical challenges associated with computing analytical Jacobian derivatives and the inherent noise sensitivity of conventional numerical methods. To this end, a validation study is conducted to (1) evaluate the estimation accuracy of the proposed IEMDO and (2) benchmark its robustness against noise in comparison with the first-order backward finite-difference method (FOBFDM). For a rigorous evaluation, a high-dimensional test matrix with a known analytical derivative is constructed. The matrix is specifically designed to emulate the dimensional characteristics of Jacobians in hyper-redundant manipulators. Specifically, we investigate a time-dependent Toeplitz matrix. According to the properties of Toeplitz matrices and the formulation, the first column is defined as

(22) $$M_{i1}\left(t\right)={\left[2+\sin\left(0.2t\right);2\sin\left(0.2t\right);\ldots ;\frac{2\sin(0.2t)}{m-1}\right]}^{T},\;i=1,\ldots ,m$$

while the first row is given by

(23) $$M_{1j}\left(t\right)=\left[2+\sin\left(0.2t\right);2\sin\left(0.2t\right);\ldots ;\frac{2\sin(0.2t)}{n-1}\right],\;j=1,\ldots ,n$$

When m≠n, the resulting matrix is an asymmetric Toeplitz matrix. We choose m=10 and n=30 to evaluate the performance of the IEMDO model in computing the derivative of high-dimensional matrices. Corresponding evaluation metrics include the Frobenius norms of the estimation error matrices $\dot{\tilde{M}}$ and $\tilde{M}$.

(24) $${∥\tilde{M}\left(t\right)∥}_{F}=\sqrt{\sum_{i=1}^{m}\sum_{j=1}^{n}{\tilde{M}}_{ij}^{2}\left(t\right)},\;{∥\dot{\tilde{M}}\left(t\right)∥}_{F}=\sqrt{\sum_{i=1}^{m}\sum_{j=1}^{n}{\dot{\tilde{M}}}_{ij}^{2}\left(t\right)}$$

To emulate real-world applications, the fourth-order Runge–Kutta method is employed with a sampling period of T=0.001s, enabling a discrete-time implementation of the proposed schemes.

The initial conditions for the simulation are set as $\hat{M}\left(0\right)\in R^{10\times 30}$, with all elements randomly generated within the interval [0,1].

The parameters for IEMDO (15) are configured as follows: γ=15, α=10, β=10, and ρ=10. The activation function chosen is the hyperbolic tangent function.

**Figure 9:**
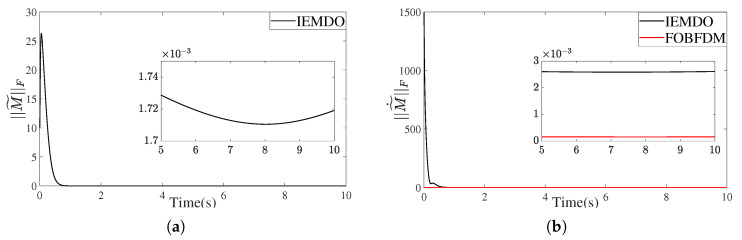
The estimation error for a high-dimensional Toeplitz matrix. (**a**) Frobenius norm of the matrix observation error. (**b**) Frobenius norm of the matrix derivative observation error.

**Figure 10:**
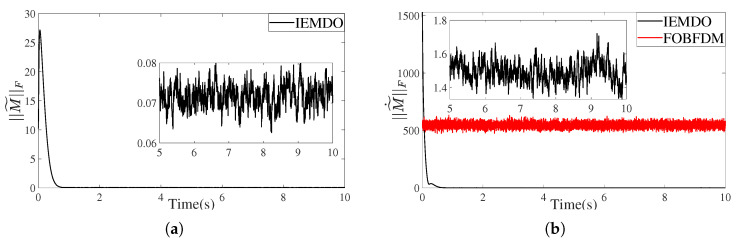
The estimation error for a high-dimensional Toeplitz matrix under noise (SNR = 30). (**a**) Frobenius norm of the matrix observation error. (**b**) Frobenius norm of the matrix derivative observation error.

Figure 9 and Figure 10 present the performance of the IEMDO and FOBFDM in estimating a high-dimensional, time-varying matrix and its derivative.

In the noise-free case (Figure 9), the IEMDO exhibits high accuracy and rapid convergence. Both the matrix estimation error $∥\tilde{M}{∥}_{F}$ and the derivative estimation error $∥\dot{\tilde{M}}{∥}_{F}$ converge to near-zero values (on the order of $10^{-3}$) within a short time, confirming the fundamental effectiveness of the observer design.

More critically, performance is assessed under a noisy condition (Figure 10, SNR = 30). The conventional numerical method, FOBFDM, is known to be highly sensitive to noise, resulting in severe oscillations in derivative estimates. This issue becomes more pronounced in practical scenarios where sampling intervals are typically 5 ms or longer, rendering traditional numerical differentiation substantially less reliable than the IEMDO. In stark contrast, the proposed observer exhibits outstanding robustness. As shown in Figure 10b, despite the presence of noise, the Frobenius norm of the derivative estimation error, $∥\dot{\tilde{M}}{∥}_{F}$, remains stable and tightly bounded. This robustness is primarily attributed to the integration mechanism and nonlinear feedback structure of the IEMDO, which effectively suppress noise and ensure smooth state estimation.

In conclusion, the IEMDO provides a practical and superior solution to overcome the ZNN controller’s critical reliance on Jacobian derivative information. Its combination of high accuracy and strong robustness makes it a reliable component for physical robotic systems, which are inevitably subject to measurement noise.

### 4.3. Trajectory Tracking Control of the Humanoid Upper-Body Robot

To validate the practical effectiveness of the proposed matrix derivative observer, a trajectory tracking experiment is conducted on the humanoid upper-body robotic platform. The problem can be formulated as Equation (1) and can be solved using the ZNN method described by Equation (2), which relies on real-time estimations of both the Jacobian matrix and its time derivative. The proposed observer is embedded into this framework to enhance estimation accuracy and ensure reliable tracking under dynamic conditions.

In the simulation, reference trajectories are defined in Cartesian space for the dual end-effectors. Each follows a circular path with a radius of 0.12 m and a period of 5 s. The centers of the circles are located at [−0.6,0.2,0.53] and [−0.6,0.2,−0.6], respectively, and both circles lie in a plane normal to the vector [1,0,0].

The parameters for the ZNN solver described by Equation (2) are set as μ=0.1, λ=50. The parameters of IEMDO in Equation (15) are the same as in the previous section.

**Figure 11:**
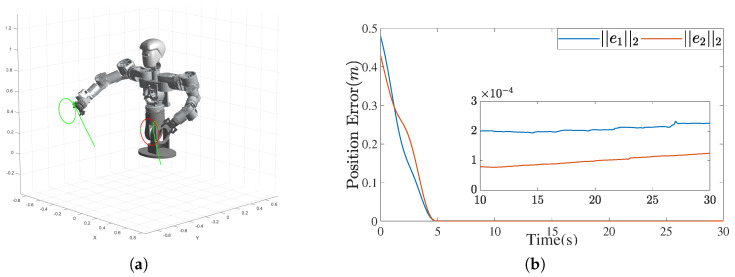
Dual-arm trajectory tracking and end-effector position error. (**a**) Simulated end-effector trajectory of the humanoid upper-body robot. (**b**) Position tracking error for both arms.

Figure 11a illustrates the 3D trajectory of the dual-arm end-effectors. In the figure, the red lines represent the desired trajectories, while the green lines show the actual trajectories. The close overlap between them demonstrates the successful tracking of the reference paths. Figure 11b shows the $\ell_{2}$-norm of position tracking errors for both arms, which rapidly converge to less than $3\times 10^{-4}$ m within 5 s and remain stable thereafter, indicating high tracking precision.

**Figure 12:**
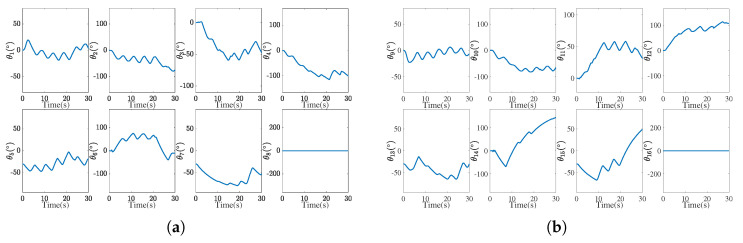
Joint trajectories of both arms solved by the IEMDO-based ZNN model. (**a**) Joint trajectories of the left arm. (**b**) Joint trajectories of the right arm.

Figure 12 presents the joint-space trajectories of both arms. Smooth and continuous profiles confirm stable control without abrupt motion, which validates the feasibility of the IEMDO-based ZNN model for practical robotic applications.

**Figure 13:**
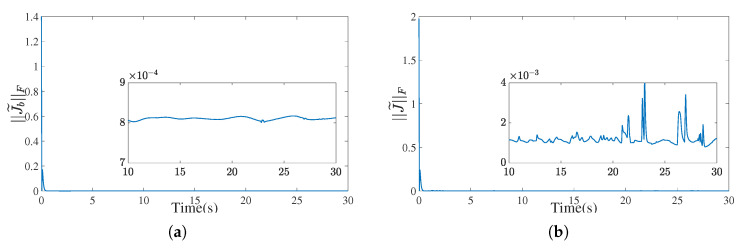
The estimation error for the Jacobian matrics solved by the IEMDO model. (**a**) Frobenius norm of the matrix $J_{b}$ observation error. (**b**) Frobenius norm of the matrix J observation error.

Figure 13 shows the Frobenius norms of the observation errors for the torso Jacobian matrix $J_{b}$ and the dual-arm Jacobian J. The error for $J_{b}$ remains below $9\times 10^{-4}$ throughout, while that for J fluctuates within $4\times 10^{-3}$. These results demonstrate the proposed observer’s ability to maintain robust estimation accuracy in the presence of dynamic variations and noise.

Overall, the simulations confirm that the integration of the matrix derivative observer significantly ensures high tracking accuracy and system robustness, making it suitable for real-time control of redundant humanoid robotic systems.

## 5. Experiment on Designed Humanoid Upper-Body Robot

To validate the effectiveness and practical applicability of the proposed IEMDO-based ZNN solver, a circular trajectory tracking experiment was conducted on the developed humanoid upper-body robotic platform. Each arm was equipped with a 3 kg payload to simulate realistic manipulation conditions. The desired end-effector trajectories of both arms were configured identically to those used in simulation, ensuring consistency between simulated and physical environments. Real-time joint position and velocity commands were executed using a Beckhoff industrial controller, enabling precise control and data acquisition throughout the experiment.

**Figure 14:**
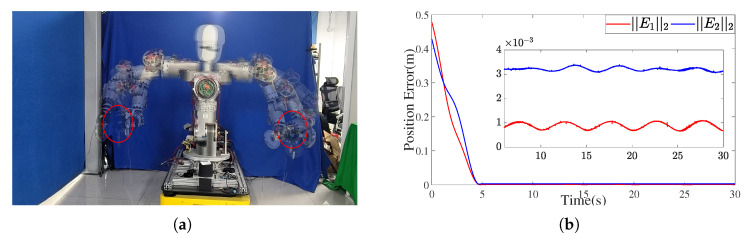
Experimental results of circular trajectory tracking. (**a**) Composite image showing dual-arm circular motion of the humanoid upper-body robot. (**b**) End-effector position tracking errors for both arms during circular trajectory execution.

The experimental results are summarized in Figure 14. Figure 14a displays a composite image capturing the robot executing synchronized circular motions with both arms. The desired trajectories are defined as two circles with radii of 0.12 m, centered at [−0.6,0.2,0.53] and [−0.6,0.2,−0.6] with a normal vector of [1,0,0] and a motion period of 5 s. Figure 14b presents the corresponding position tracking errors for both end-effectors, obtained by comparing measured trajectories calculated from joint angle sensors with the desired paths.

The results confirm that the IEMDO-enhanced ZNN controller achieves high tracking accuracy, with the steady-state position error of the left arm maintained at around 1 mm and the steady-state position error of the right arm maintained at 3 mm. This minor residual error is likely attributable to unmodeled dynamics in the physical system, such as joint friction and backlash in the harmonic drives, yet the overall performance robustly demonstrates the practical applicability and precision of the proposed control framework.

## 6. Conclusions

In this study, a 22-DOF humanoid upper-body robotic system was developed, inspired by the biomechanics of the human sternoclavicular joint, torso, and head. The system comprises dual 8-DOF arms, a 3-DOF torso, and a 3-DOF head, all constructed using standardized hollow joint modules. To enhance modularity and signal reliability, a low-cost resolver-based joint design was adopted, offering strong anti-interference capability and compact integration suited for high-DOF robotic architectures. Comprehensive workspace analysis demonstrates that the proposed configuration significantly outperforms conventional dual-arm platforms in both total and common workspace volumes, underscoring its superior spatial adaptability and coordination potential. To address the trajectory tracking problem under joint angle and velocity constraints, a ZNN-based controller was enhanced by introducing an integral-enhanced matrix derivative observer (IEMDO), effectively eliminating the dependence on prior Jacobian matrix derivatives. Simulation results confirm the high estimation accuracy and noise robustness of the proposed observer in high-dimensional scenarios. Furthermore, both simulations and physical experiments validate that integrating IEMDO into the ZNN solver ensures high end-effector trajectory tracking accuracy. The presented framework thus offers a practical and theoretically grounded approach to trajectory control for redundant humanoid manipulators.

Future work will focus on leveraging the robot’s unique kinematic advantages—namely its expanded common and total workspace—in complex, real-world environments. We plan to evaluate its performance in unstructured household scenarios, addressing tasks such as retrieving objects from deep shelves and performing precise cleaning operations in cluttered spaces. In addition, its applicability to industrial assistance will be explored, where the ability to reach into machinery and confined workspaces without repositioning the base could substantially enhance both task efficiency and human safety.

## Figures and Tables

**Table 1:** Comparison of DOF allocations in representative humanoid robots.

Robot	Head DOFs	Torso DOFs	Arm DOFs
Robonaut2 [5]	3	1	7
Justin [6]	2	3	7
HUBO [7]	1	1	8
ARMAR 6 [8]	2	1	8
HRP-5P [9]	2	3	9
SURENA IV [10]	3	2	7
BHR-6 [11]	2	3	4
JET [12]	2	2	7
TOCABI [13]	2	1	7
BIT-DMR [14]	2	0	7
FC-EODR [15]	2	1	7
iCub 3 [16]	3	3	7

**Table 2:** Denavit–Hartenberg (D-H) parameters of the designed humanoid upper-body robot.

Joint $\theta \left(i\right)\;{(}^{\circ })$	Offset of $\theta \left(i\right)\;{(}^{\circ })$	$\alpha_{i}\;{(}^{\circ })$	$a_{i}\;\left(m\right)$	$d_{i}\;\left(m\right)$	Range (°)
$\theta_{b}\left(1\right)$	180	90	−0.0155	0	−30∼30
$\theta_{b}\left(2\right)$	−90	90	0.377	0	−135∼135
$\theta_{b}\left(3\right)$	180	90	0.0345	±0.13	−10∼10
θ(1)/θ(9)	90	90	0.1445	0	−80∼80
θ(2)/θ(10)	90	90	±0.3235	0	−160∼160
θ(3)/θ(11)	90	90	0	0	−110∼23
θ(4)/θ(12)	90	90	−0.2662	0	−165∼165
θ(5)/θ(13)	90	90	0	0	−95∼95
θ(6)/θ(14)	90	90	−0.2482	0	−180∼180
θ(7)/θ(15)	180	90	0	0	−95∼95
θ(8)/θ(16)	0	0	−0.345	0	−360∼360

**Table 3:** Workspace analysis results.

Configuration	DOFs	Total $\left(m^{3}\right)$	Common $\left(m^{3}\right)$	PTCWA $\left(m^{6}\right)$
1	22 DOF	10.754	5.3398	57.4242
2	20 DOF	10.6714	3.6937	39.4169
3	19 DOF	6.2286	2.3854	14.8577
4	17 DOF	5.7297	1.1403	6.5335

## Data Availability

The original contributions presented in this study are included in the article or Supplementary Material.

## Supplementary Materials

The following supporting information can be downloaded at: https://www.mdpi.com/article/10.3390/biomimetics10080505/s1.
